# A model of wild bee populations accounting for spatial heterogeneity and climate‐induced temporal variability of food resources at the landscape level

**DOI:** 10.1002/ece3.9014

**Published:** 2022-06-17

**Authors:** Maria Blasi, Yann Clough, Anna Maria Jönsson, Ullrika Sahlin

**Affiliations:** ^1^ Centre for Environmental and Climate Science Lund University Lund Sweden; ^2^ Department of Physical Geography and Ecosystem Science Lund University Lund Sweden

**Keywords:** agricultural landscape, drought, land‐use, phenology, pollination services, wild bees

## Abstract

The viability of wild bee populations and the pollination services that they provide are driven by the availability of food resources during their activity period and within the surroundings of their nesting sites. Changes in climate and land use influence the availability of these resources and are major threats to declining bee populations. Because wild bees may be vulnerable to interactions between these threats, spatially explicit models of population dynamics that capture how bee populations jointly respond to land use at a landscape scale and weather are needed. Here, we developed a spatially and temporally explicit theoretical model of wild bee populations aiming for a middle ground between the existing mapping of visitation rates using foraging equations and more refined agent‐based modeling. The model is developed for *Bombus* sp. and captures within‐season colony dynamics. The model describes mechanistically foraging at the colony level and temporal population dynamics for an average colony at the landscape level. Stages in population dynamics are temperature‐dependent triggered by a theoretical generalized seasonal progression, which can be informed by growing degree days. The purpose of the LandscapePhenoBee model is to evaluate the impact of system changes and within‐season variability in resources on bee population sizes and crop visitation rates. In a simulation study, we used the model to evaluate the impact of the shortage of food resources in the landscape arising from extreme drought events in different types of landscapes (ranging from different proportions of semi‐natural habitats and early and late flowering crops) on bumblebee populations.

## Graphical abstract

A theoretical ecological model for pollinators and pollination, with climate and land use inputs that will influence population dynamics and ecosystem services delivery. In a simulation study, we demonstrate the model’s ability to model populations in different landscapes and to detect impacts from the shortage of food resources occurring from extreme drought events.

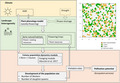

## 1 INTRODUCTION

Bees are key pollinators in many natural ecosystems and provide pollination services for agricultural crops (Klein et al., 2007). However, domestic and wild bees are threatened by consequences of climate change and land‐use change (IPBES, 2016; Soroye et al., 2020; Vanbergen & the Pollinators Initiative, 2013) and have been negatively affected in many parts of the world, at the local and regional scale (Biesmeijer et al., 2006; Potts et al., 2010). A good quantitative understanding of global change effects on pollinators is important to understand the consequences for pollination services and the need for conservation. Pollinator models are increasingly used in ecosystem service mapping and impact assessments to relate land‐use patterns to the population size of pollinators such as bees (Becher et al., 2018; Gardner et al., 2021; Koh et al., 2016). The consideration of variability in weather and climate has received less attention, despite its importance in driving demographic rates, population sizes (Selwood et al., 2015), and distributions (Aguirre‐Gutiérrez et al., 2017). Indeed, land use and climate change have interactive effects on pollinators (Oliver et al., 2015; Prestele et al., 2021) and thus should be considered jointly.

Climate change effects include gradual changes in annual means, altered seasonal variation, and increased frequency of extreme events, such as heatwaves and extended drought periods (IPCC, 2021). The phenologies of many ecological processes are moderated by temperature and thus sensitive to climate change. For example, plant phenology shifts can potentially affect interactions between pollinators and host plants, due to mismatches of flowering and pollinator foraging (when shifts are asynchronous) (Freimuth et al., 2022; Gérard et al., 2020; Memmott et al., 2007; Scaven & Rafferty, 2013). Under drought conditions, floral resources are reduced for pollinators, affecting pollinator survival (Phillips et al., 2018; Wilson Rankin et al., 2020).

Bumblebees (genus Bombus) contribute critically to the provision of crop pollination (Table S2 from Kleijn et al., 2015). The presence of floral (i.e., semi‐natural habitats (SNH) and flowering crops) and nesting resources (i.e., natural and SNH, field edges, forests, meadows, and permanent grasslands) in the landscape during their life span is key for their colony development. This includes among others the ability to produce large foragers (Persson & Smith, 2011), to produce males and new queens at the end of the season (Rundlöf et al., 2014), and to survive during winter (Persson & Smith, 2013). Intensive agricultural management in large arable fields reduces the availability of nesting sites, with reduced crop diversity (Aizen et al., 2019) being associated with the dominance of individual flowering crops which may cause bottlenecks in terms of foraging resources for pollinators outside of the flowering period of these crops. Understanding how spatial and temporal variability of resources driven by land use and climate change interact at the landscape level and affect pollinator populations is crucial to help ensure that crop demands and pollinator supplies are well‐matched (Settele et al., 2016). Bees are affected by climate change and there are adaptive limits of this pollinator group to track climate change (Kerr et al., 2015; Potts et al., 2010; Prestele et al., 2021; Sirois‐Delisle & Kerr, 2018). While the availability of habitats rich in resources for bees, such as SNH, may be able to offset the deleterious effects of climate change on bee communities (Papanikolaou et al., 2017), the interactions between the effects of land use and climate are still poorly studied.

Spatially explicit models of pollinators produce bee visitation rates from proxies of bee abundance and floral resources at a landscape scale, which is used as a representation for the supply of pollination (e.g., InVEST pollination module using Lonsdorf et al., 2009). Visitation rates can be derived from central place foraging theory (Lonsdorf et al., 2009; Olsson et al., 2015), assuming that fitness is entirely dependent on the distribution of floral resources around the nest as derived from a spatially explicit land use map. These models produce indices of bee visitation rates for fixed floral resources dividing the flying season into two (Häussler et al., 2017) or three periods (see Gardner et al. (2020)). This allows for a limited variability in resources or visitation rates within and between seasons, and therefore makes it difficult to study more fine‐scale variability in floral resources driven by climate (e.g., the different start of flowering). Interactions between pollinators and land use, and thereby changes in nesting and floral resources, require models combining foraging theory with population dynamics. For these models to be sensitive to variability in resources in the landscape, there is a need to model interactions at relevant spatial and temporal scales and include changes in growing conditions due to climate conditions (Johansson & Bolmgren, 2019).

To fill this gap, we developed a spatially and temporally explicit model for wild bees, referred to from now on as the LandscapePhenoBee model, that uses the foraging function of an existing pollination model developed by Häussler et al. (2017). LandscapePhenoBee can be used to explain and project population dynamics based on changes in colony dynamics driven by landscape components and weather‐induced variability in resources. The landscape components consider variation in landscape‐scale land use covers both differences in composition (proportions of different habitat types) and configuration (e.g., field sizes). Weather‐induced variability of resources entails that the phenological growth of plant resources is triggered by a generalized seasonal progression, but also that extreme weather events (e.g., droughts), can influence the growth of resources. The different growth development of different stages at the colony level is also triggered by a theoretical generalized seasonal progression.

The aim of this study is to use the LandscapePhenoBee model to explore the effect of the temporary, drought‐induced shortage in food resources on the population viability of bumblebees, evaluated by (1) population size and (2) production of queens, and on (3) the pollination services provided by bumblebees in different types of landscapes, ranging from simple (landscapes with a low proportion of SNH) to complex (landscapes with a high proportion of SNH) agricultural landscapes, and including early and late flowering crops. We expect that (1) bumblebee populations to be more severely affected by drought in less complex landscapes since the distance between a nest and floral resources is on average larger in a simple compared to complex landscapes and (2) the presence of early flowering crops will have an effect on the colony dynamics, expecting higher production of bumblebee workers with landscapes with lots of early flowering crops, while landscapes with more late‐flowering crops will have a positive effect on the production of queens. The influence of the population parameters is evaluated by sensitivity analysis.

## 2 METHODS

### 2.1 Theoretical model description

The LandscapePhenoBee model is designed to simulate wild bee species that are central place foragers, for example, a bumblebee species. We considered a fictive common early active bumblebee that stands for several species that are known to be important crop visiting species, including *Bombus terrestris, Bombus lucorum,* and *Bombus lapidarius* (Kleijn et al., 2015).

**FIGURE 1:**
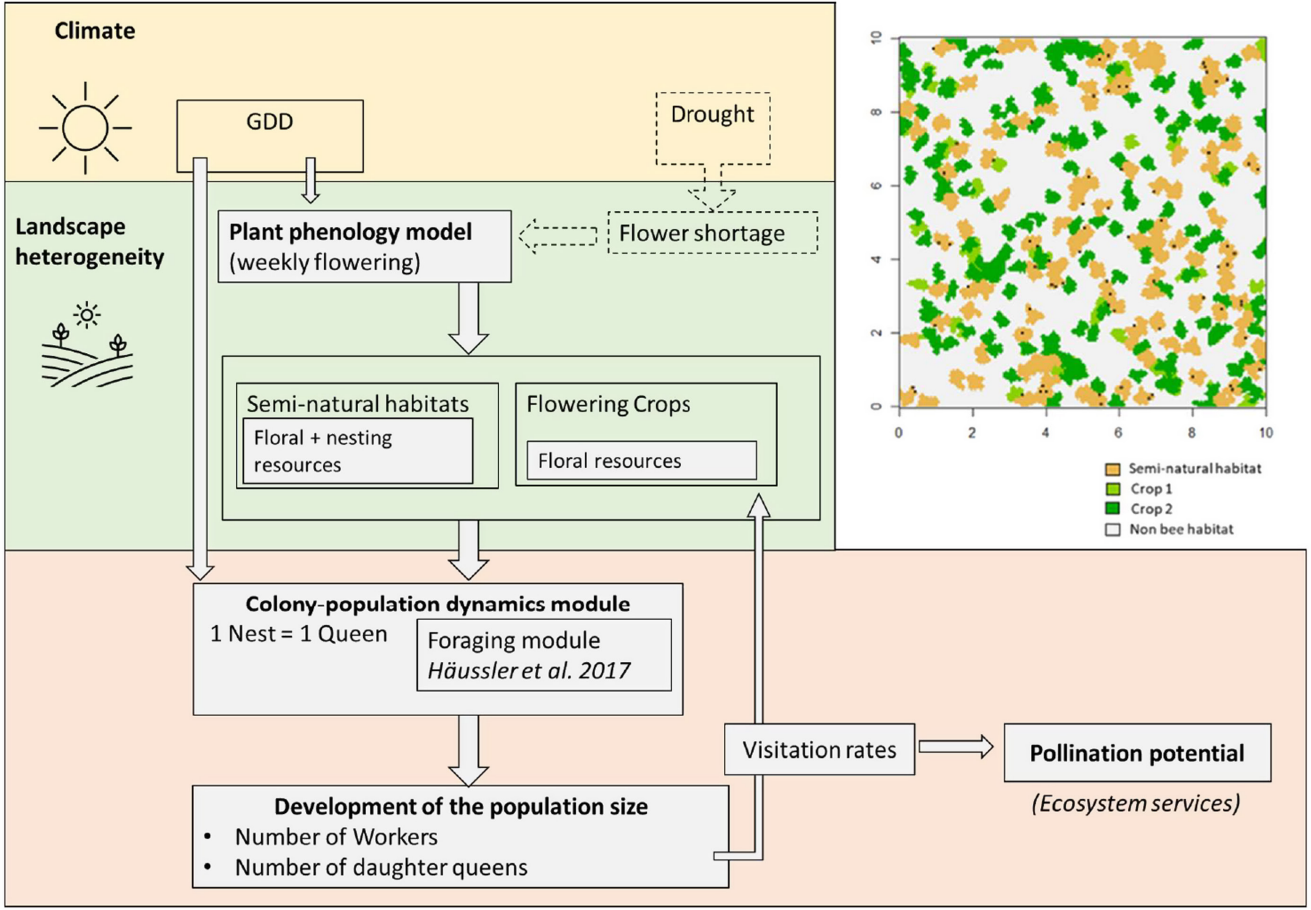
Representation of the spatial–temporal explicit model developed in this study including the climate and landscape inputs and the outputs for the wild bee population dynamics and the ecosystem service. The simulated landscapes include four different types of patches: SNH, early flowering crop, late‐flowering crop, and non‐bee habitat (habitat that does not provide any resources for bees)

The model consists of three parts: a phenological model, the foraging module, and the colony‐population dynamics module (Figure 1). The phenological model simulates the availability of weekly floral resources through the season/year (see Section 2.1.2). The foraging module builds on an existing bee foraging model developed by Häussler et al. (2017) but expresses visitation rates with a higher time resolution (per week, instead of two time periods) and focuses on the colony and population dynamics at the landscape scale within the year (instead of as in Häussler et al. between years) (see Table S1 with a more extended comparison between the two models). The population dynamics are expressed for an average population in the landscape, which is informed by the total amount of resources gathered by nests in the landscape. The model produces the development of the population size and pollination services in the total landscape as outputs. Input and output model parameters are described in the Tables S1 and S2.

#### 2.1.1 Spatially explicit resources in the landscape

Spatial variability in nesting and floral resources is represented by spatially explicit maps with a resolution of 10 × 10 m. The size of a landscape (2010 × 2010 m) was considered to be sufficiently large to account for bee foraging distance, which is normally about 1000 m or less for bumblebees (Osborne et al., 2008). The landscape consists of three types of habitat patches: early‐ and late‐flowering crops, and SNH. The rest of the grid consists of a matrix of non‐suitable habitats for the bees (representing for example non‐flowering arable land, or sealed urban areas), from this point forward referred to as non‐bee habitat. The resolution, landscape size, and categorization of habitats can be altered.

Semi‐natural habitats are the only habitats (among those represented) where nesting is considered possible. The nests are assigned randomly in the landscape, and the density of nests in the landscape is determined by the nest density parameter. The nest allocation is fixed for a given season. We specified that each SNH cell can have either no nests or one nest (but this can be changed if the model runs in a different resolution). The total number of nests in a landscape is proportional to the amount of SNH in the landscape (the more SNH in the landscape, the more nests).

We created artificial landscapes (e.g., Figure 1) with different proportions and configurations of four land‐use classes (SNH, early and late flowering crop, and non‐bee habitat) using the R package *landscapeR* (Masante, 2016; Thomas et al., 2020). The number and size of the patches of SNH were used to randomly create landscapes with different proportions of SNH between 5% and 25% which was used to describe spatial heterogeneity between simple and complex landscapes (Holzschuh et al., 2016; Scheper et al., 2014). The average size of crop patches was set to 500 m^2^, and the proportion of flowering crops was set such that these constitute together with SNH, 60% of the area in the landscape.

Within the proportion of crops, fields of early‐ and late‐flowering crops (MFC) were randomly assigned to achieve a certain proportion of early versus late. Consequently, the presence of these two crops was negatively correlated (the more early‐flowering crop, the less late‐flowering crop).

Temporal variability in floral resources is considered by mapping floral resources with a weekly temporal resolution based on the floral phenology model (see Section 2.1.2). The floral phenology model provides the start, peak, and end of flowering in each habitat type based on simulated growing degree days (GDD) such that for each week *t*, cell *i* has the floral resource value *F*(*t*,*i*) (Figure 1, and see Figure S1).

#### 2.1.2 Floral phenological model

The influence of climate on population dynamics and temporal variability in the availability of floral resources for different land resources during the year is considered by linking the LandscapePhenoBee model to a theoretical generalized seasonal progression from 0 to 100, mimicking a sigmoid function of cumulative GDD for northern hemisphere context (see Figure S4). If daily observed temperatures are available, the model allows for a simple calculation of GDD and uses it as model input. In this manuscript, we present a seasonal progression with an arbitrary GDD.

We modeled the temporal dynamics in floral resources for each land use separately as a function of the generalized seasonal progression following a sigmoid function product of a cumulative standard normal distribution and defined for day *y* at time *t* as:

(1) $$\Phi \left(\frac{y\left(t\right)-200}{65}\right)\cdot 100$$

where Φ is the probability function for the normal distribution (Figure S4). For each land use type *h*, the start and end of floral resources are given by a theoretical $\mathrm{gd}d_{\text{start},h}$ and $\mathrm{gd}d_{\mathrm{end},h}$ The floral resources in cell *i* at time *t* is

(2) $$F\left(t,i\right)=\sum_{h}\left(1-4*{\left(z_{h}\left(t\right)-0.5\right)}^{2}\right)*f_{\max,h}$$

where $z_{h}\left(t\right)$ is a standardized value between 0 and 1 of the day of the year corresponding to time *t*, derived by the following expression
(3)

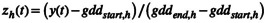




The maximum floral resources in habitat *h*, $f_{\max,h}$, is a theoretical parameter that has assigned a value between 0 and 1 to capture how the floral resources in different habitats relate to each other, chosen to have the following relations: early MFC > late MFC > SNH. The reason was to simulate the development of resources that these different habitats provide along the season, a peak of resources early in the season by early MFC, a lower peak of resources later in the season by late MFC, and lower but constant floral resources provided by SNH (see also Figure 3a).

#### 2.1.3 Bee foraging

The number of foraging bees from the nest in cell *i* at time *t* is *X*(*t*,*i*). The foraging bees are initially overwintering queens, that is, *X*(*t*,*i*) is 1, and later workers are produced by the queens. The foraging function is an exponential kernel reweighted by the floral resources (Häussler et al., 2017). The rate at which each cell *j* is visited by foraging bees from cell *i* during week *t* is:

(4) $$VR_{i,j}^{t}=X\left(t,i\right)\frac{F\left(t,j\right)e^{-d\left(i,j\right)/\gamma }}{\sum_{u\in U_{i}}F\left(t,u\right)e^{-d\left(u,j\right)/\gamma }}\;$$

where *F*(*t,j*) is the floral value of cell *j, d*(*i,j*) is the Euclidean distance between cells *i* and *j, γ* is the mean dispersal distance when foraging; *U*_*i*_ is the set of cells reachable from cell *i*. The denominator in Equation 4 weighs the attractiveness of cell *i* compared to the total attractiveness of the cells in the landscape and by foraging distances. In this way, a cell further away from cell *j* compared to cell *i* but with higher floral resources compared to *i* can be receiving more visits. Thus, the resources collected correspond to the distance‐weighted resource values from cells in which bees are nesting. The spatial layer is treated as a toroid, that is, the edges are connected to each other such that a bee moving beyond the boundary of the spatial layer appears at the opposing edge.

Under the assumption that there is no depletion of floral resources in the landscape, the resources collected by foragers emerging from the nest in cell *i* at time *t* is

(5) $$r\left(t,i\right)=\sum_{j\in U_{i}}F\left(t,j\right)VR_{i,j}^{t}$$

where *U*_*i*_ are the cells within reach from cell *i*.

The average amount of resources gathered per nest in the landscape at time *t* is

(6) $$R\left(t\right)=\frac{\sum_{i=1}^{N}r\left(t,i\right)}{N}$$

#### 2.1.4 Bee colony and population dynamics

The model covers the active period of bees in the season for a given year, that is, from the emergence of queens in the spring until the production of daughter queens at the end of the summer.

**FIGURE 2:**
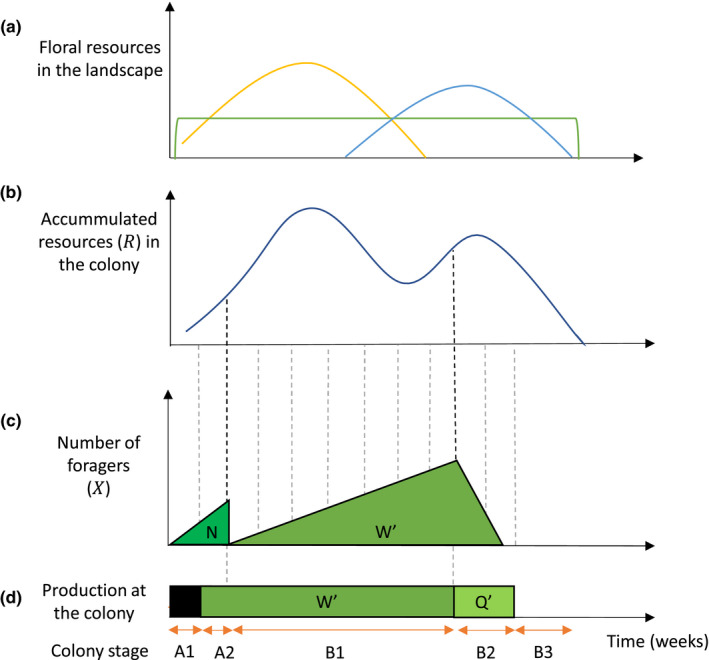
Graphical representation of the modeling approach between the landscape floral resources and the link with population dynamics. Floral resources in the landscape in time (a), represented by early flowering crop (yellow), late‐flowering crop (blue), and SNH (green); the accumulated resources in the bee colony (b); the number of foragers (c); and (d) the bee caste produced at the colony according to the different stages of the colony through the season: A1, Overwintered queens foraging, and workers not yet being produced. A2, Overwintered queens foraging and workers are produced. B1, Workers are foraging and being produced. B2, Workers are foraging and daughter queens are being produced. B3, No new individuals are produced, and workers and daughter queens start to decay. N stands for nests (1 nest, 1 queen), W′ are workers and Q' are daughter queens

Bee population dynamics are modeled as two main stages within a season, corresponding to who is foraging: either overwintering queens (1) or workers (2) (see Figure 2). In turn, these two stages are subdivided into stages depending on what is being reproduced:

Stage A1: Overwintered queens are foraging, and workers are not yet being produced.

Stage A2: Overwintered queens are foraging, and workers are produced.

Stage B1: Workers are foraging and being produced.

Stage B2: Workers are foraging, and daughter queens are being produced.

Stage B3: No new individuals are produced, and the mortality of workers and daughter queens increases.

Queens emerge when theoretical GDD reaches φ (Table S2, Figure S4) and start producing workers after 2 weeks (from A1 to A2). The number of foragers during stage A1 and A2 are one per nest, that is, *X*(*t*,*i*) = 1 if there is a nest in cell *i*, and 0 otherwise.

In stage A2, the average number of workers per nest at time *t*, *W*(*t*), is given by:

(7) $$W\left(t\right)=\delta_{t}W\left(t-1\right)+w\left(t\right)$$

where *δ*_*t*_ < 1 is the survival of the workers from time *t*−1 and *w*(*t*) is the average number of workers produced per nest at time *t*. Growth depends on the resources gathered during the two previous time periods (*t*−1 and *t*−2) according to a plateau function:

(8) $$w\left(t\right)=\alpha_{t}\left(1-\exp\left(-\frac{R\left(t-1\right)+R\left(t-2\right)}{2\beta_{t}}\right)\right)$$

where α_t_ is the maximum number of workers that can be produced per nest at time *t* and *β*_t_ is a parameter determining for which amount of resources half of the potential number of workers are being produced at time *t*. Survival rate and the two growth parameters are constant values *δ*, *α*, and *β*, respectively, during stages A2–B2.

Workers take over the foraging, that is, there is a transition from stages A2 to B1, when a theoretical GDD has reached *μ* (where *μ* > φ) (Table S2, Figure S4). During this period the number of foragers emerging from nest *i* is *X*(*t*,*i*) = *pw W*(*t*).

The colonies begin production of daughter queens, that is, the transition from B1 to B2, when theoretical GDD reaches *ψ* (where *ψ* > *μ*) (Table S2, Figure S4). The queen produces new workers and daughter queens (stage B2), with the proportions ϵ: (1 – ϵ). The number of workers in the landscape at the time *t* is

(9) $$W\left(t\right)=\delta_{t}W\left(t-1\right)+\epsilon w\left(t\right)$$

and the number of queens at the time *t* is

(10) $$Q\left(t\right)=\delta_{t}\;Q\left(t-1\right)+\left(1-\epsilon \right)w\left(t\right)$$

No more individuals are produced, that is, *w*(*t*) = 0 (stage B3), when no resources have been gathered for two consecutive time periods. This is set to occur when both *R*(*t*−1) and *R*(*t*−2) are close to zero. During this stage, *δ*_*t*_ is set to decrease with time resulting in a declining survival at the end of the season.

#### 2.1.5 Pollination potential

Pollination potential at time *t, P*_*t*_ is a function of the total visitation rate per floral resource in crops according to

(11) $$P_{t}=\sum_{i}F\left(t,i\right)\left(1-\exp\left(-\kappa \frac{\sum_{j\in U_{i}}VR_{j,i}^{t}}{F\left(t,i\right)}\right)\right)\;$$

where *κ* is a parameter adjusting how quickly the visitation rate per crop floral resource reaches the maximum pollination potential. The total pollination potential is a score $\mathrm{PS}=\sum_{t}P_{t}$ that represents how much the crops can benefit from pollination visits during a season at the landscape scale. The pollination potential is calculated for the crop habitats of the landscape.

The model assumes that there is no depletion of floral resources—the effect that pollination might have on floral resources—an assumption shared by other pollinator models Lonsdorf et al. (2009), Olsson et al. (2015), and Häussler et al. (2017) (but considered in BumblebeeHAVE [Becher et al., 2018]). However, the model controls for resource competition by defining that the average amount of floral resources collected per nest depends on the number of nests in the landscape. Therefore, population growth is density‐dependent, such that for a fixed amount of floral resources in a landscape with more nests, there are fewer resources collected by the colony, and a lower number of workers or queens can be produced.

#### 2.1.6 Model outputs

The model outputs for the population dynamics include the population size approximated by the maximum number of workers at the peak of population growth (MaxW), the total number of daughter queens produced at the end of the season (TQ), and the pollination potential per season in the landscape (PS) (Table S3). MaxW and TQ are summarized at the nest level, which is done by aggregating over nests in the landscape and divided by the number of nests (in the landscape).

### 2.2 Model implementation

#### 2.2.1 Drought

**FIGURE 3:**
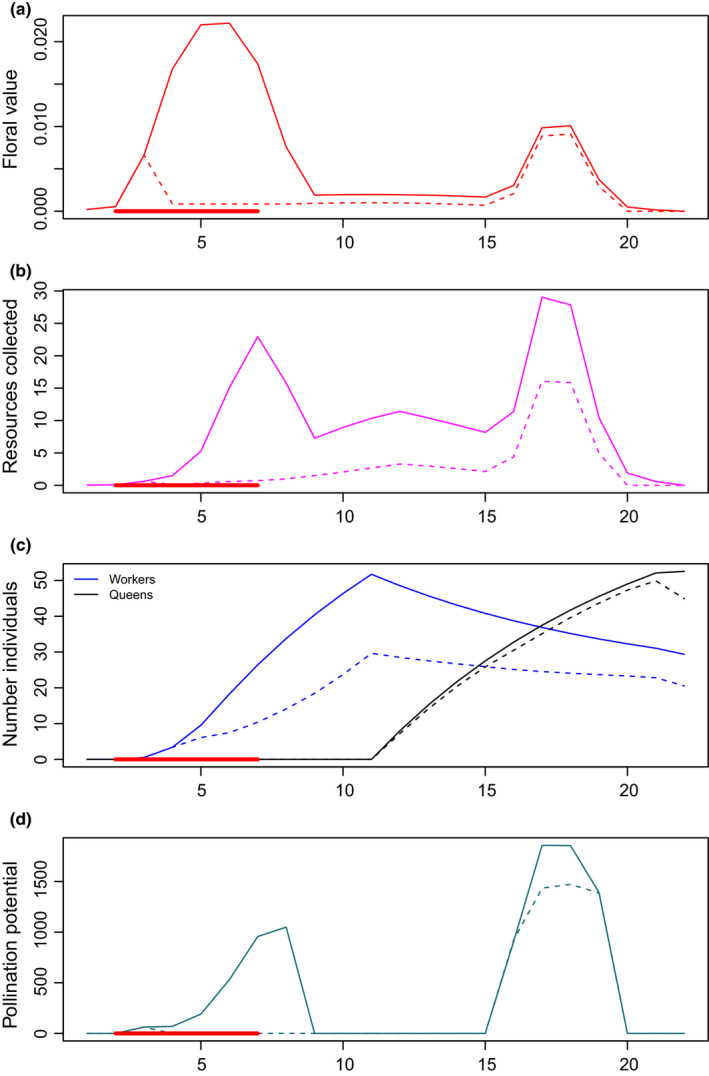
One example of graphical representation of the model simulation for the floral resources in the landscape along the season (a); the resources gathered at the colony (b); the number of workers and queens (c); and the pollination potential (d). All graphs include also the simulations under drought conditions, indicated with a dashed line. The red segments in the graph indicate when the drought takes place

In this simulation study, we introduced a drought event as a reduction in floral resources that was defined by a fixed starting point early in the season and had a duration between 1 and 4 weeks (see Figure 3, Figures S2 and S3). The reduction of floral resources was simulated in a way that penalizes the growth of resources: if in normal conditions the growth is positive, the growth in drought conditions will be close to zero, while if the growth is negative, under drought conditions this will translate in a 50% larger reduction of growth. Drought and no‐drought conditions were evaluated as a case–control setup, which allowed us to study the effect of drought by comparing the model results with and without drought while the rest of the model design, including the generated landscape and distribution of floral resources in space, was kept constant within case–control pairs. The start of the drought was defined by an arbitrary GDD above 5°C, which translates into a corresponding day of the year, following the other events triggered by arbitrary GDD (see Figure S4).

#### 2.2.2 Simulation design

To study the effect of landscape heterogeneity on the impact of drought on bee populations and their pollination, a simulation experiment was constructed by varying the amount and size of patches of SNH, the proportion of early and late flowering crop habitat, and the duration of drought. We generated a simulation design for landscapes using latin hypercube sampling of the following factors: SNH sizes within the range of 5000–15,000 m^2^, the proportion of SNH in the landscape between 5% and 25%, and duration of drought between 1 and 4 weeks. The proportion of SNH in the landscape would determine the amount of MFC in the landscape, and the proportion of early and later MFC was defined as described in Section 2.1.1.

We applied 5 iterations of 20 sampling combinations, generating 100 unique landscapes. For each draw of design variables (iteration), a landscape and associated floral resources were generated (first without and then with drought for the given duration), obtaining a total sample size of 200 simulations. Landscape heterogeneity and the actual proportion of SNH were calculated from the artificial landscape. The model and simulation have been implemented in R version 4.1.1 (R Core Team, 2021).

### 2.3 Analysis

#### 2.3.1 Regression analysis

Using the *lmer* function in the R package lme4 (Bates et al., 2014), we fitted separate linear mixed models for the response variables: maximum number of workers per nest (MaxW), the total number of daughter queens produced per nest (TQ), and the pollination potential (PS). In each model, we included the explanatory variables drought, proportion of SNH, and the proportion of early‐flowering crops in the landscape (including a linear and a quadratic term [i.e., second‐order polynomial]), as well as the interactions between landscape variables and drought as explanatory variables. Models included the landscape identification number as a random factor (to be able to evaluate the case–control setup with the drought). We assessed the significance of the main effects using likelihood‐ratio tests comparing models with and without the effect (Table S5).

#### 2.3.2 Sensitivity analysis

The influences of the population model parameters were evaluated with respect to the estimated effects of SNH, early flowering crop, and drought, on the three outcome quantities (MaxW, TQ, and the PS). We, therefore, built a separate model for each outcome quantity and included the mentioned model predictors without interactions. We used a local sensitivity analysis, where parameters are varied ±10% around their nominal value one at a time, keeping all the other parameters fixed. Sensitivity was quantified by a score S, defined as the slope parameter in a linear regression of the percent change in the parameter values against the effect sizes, divided by the estimated effect size for the nominal parameter value.

The parameters *fmax,h* were not used in the sensitivity analysis because these characterize the input to the population model and are therefore not parameters of the population model as such, which was the target of the sensitivity analysis in the study. The nesting density parameter was included in the sensitivity parameter because it is used to derive the initial population size and can therefore be seen as a parameter of the population model.

## 3 RESULTS

### 3.1 Regression analysis

**FIGURE 4:**
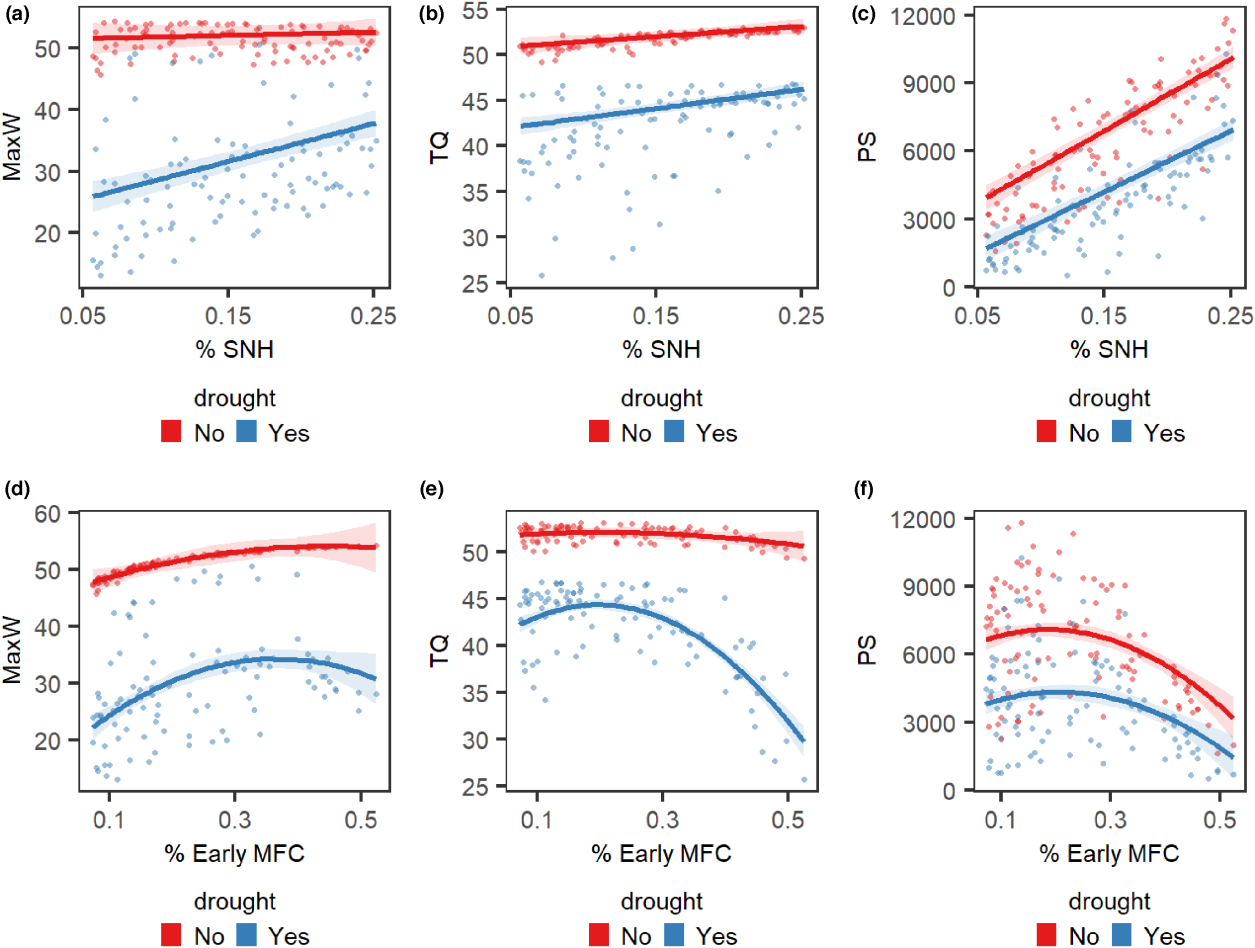
Predicted values (marginal effects) for each model terms' interactions. The interactions are the effect of drought and the amount of semi‐natural habitats (SNH) (a–c), and the effect of drought and early‐flowering crop (d–f) in the landscape for the maximum number of workers produced per week (MaxW) (a, d), the number of daughter queens (TQ) (b, e), and the pollination potential (PS) (c, f)

All three output quantities were significantly affected by the proportions of SNH and MFC and if there is a drought or not (*p*‐values for all LR statistics were <.01, see Table S8). The drought had a negative impact on all output quantities. The negative effect of drought on the number of workers and queens produced was reduced at higher levels of SNH (Table 1, Figure 4). The proportion of SNH had a positive linear effect on pollination potential and queen production under both drought and no drought conditions, but on population size only during drought conditions (Table 1, Figure 4a–c). The proportion of early‐flowering crops (by design negatively correlated with the proportion of the late‐flowering crops) had a positive effect on population size (Figure 4d), and a negative effect on queen production and pollination potential (Figure 4e,f). The relationship between the proportion of early‐flowering crops and pollination potential was best described by a quadratic model. Under drought conditions, the number of queens produced was reduced when there were a lot of early‐flowering crops, and thus a lower cover of late‐flowering crops (number of queens ranging from 33 to 46 between the landscapes). The maximum number of workers produced per week was less variable under no drought conditions (numbers ranging from 46 to 54, affecting the average total number of workers produced by the colony—550–670) compared to drought conditions (numbers ranging from 14 to 52, affecting the average total number of workers produced by the colony—186–638). To give an example of how the initial conditions relate to model outputs, in a landscape with 10% of SNH, there were assigned randomly 49 nests given the parameter nest density, therefore 49 colonies with one bumblebee queen in each. Based on these initial nesting conditions, the population model calculated that each colony produced on average 585 workers during all seasons, with a maximum number of 49 workers produced per week. However, in the same landscape under drought conditions, there were produced 219.97 workers, with a maximum number of 16.47 per week.

**TABLE 1:** Linear mixed‐effect model results describe the effects of landscape characteristics (% SNH and early MFC, represented as MFC) and effects of drought on the pollination potential (PS), the maximum number of workers produced per week (MaxW), and the total number of daughter queens (TQ)

Predictors	MaxW				TQ				PS			
	Estimates	*SE*	*df*	*p*	Estimates	*SE*	*df*	*p*	Estimates	*SE*	*df*	*p*
(Intercept)	44.21	2.59	199.88	**<.001**	49.86	0.90	195.63	**<.001**	1483.40	547.80	146.10	**.008**
SNH%	3.33	8.71	199.88	.703	11.65	3.02	195.63	**<.001**	31,387.20	1843.00	146.10	**<.001**
MFC%	42.90	19.64	199.88	**.030**	2.86	6.80	195.63	.675	9320.80	4155.10	146.10	**.026**
I(MFC%)^2^	−50.73	37.44	199.88	.177	−6.50	12.96	195.63	.617	−26,194.50	7921.70	146.10	**.001**
Drought	−37.89	3.62	100.00	**<.001**	−11.14	1.17	100.00	**<.001**	−2161.50	485.60	100.00	**<.001**
SNH % × drought	86.14	12.17	100.00	**<.001**	12.50	3.93	100.00	**.002**	−3236.00	1633.70	100.00	**.050**
MFC % × drought	−15.86	27.43	100.00	.564	7.76	8.87	100.00	.384	−4954.20	3683.30	100.00	.182
I(MFC %)^2^ × drought	87.71	52.30	100.00	.097	−27.61	16.91	100.00	.106	15,133.90	7022.30	100.00	**.034**

*Note*: Model estimates, standard error (*SE*), degrees of freedom (*df*), and *p*‐values (*p*). Significant *p*‐values (<.05) are shown in bold. A sample size of the simulated data is 200 (resulting from 100 unique generated landscapes applied with both drought and no drought conditions). All models include landscape identification numbers as a random factor. All models presented *R* ^2^ >.8.

### 3.2 Sensitivity analysis

The sensitivity analysis showed that the parameter survival rate (δ) had the highest influence on the three output quantities (Figure S5) as well as estimated effects from %SNH and % Early flowering crop and Drought (Figure S6). The parameter, temperature‐dependent timing for the start of queen production (*ψ*), and the two growth rate parameters (*α* and *β*) also had a high influence on the model outcome. The nesting density and proportion of workers foraging (*pw*) had a minor influence. The parameters, arbitrary GDD, for workers to start foraging (*μ*), the proportion of workers produced compared to new queens (*ε*), and arbitrary GDD for queen emergence (*φ*), had the least influence on the estimates of the effects of SNH and early flowering crop and drought on all three output quantities. See Tables S4–S7 and Figures S5–S9 for the results of the sensitivity analysis).

## 4 DISCUSSION

The LandscapePhenoBee model simulates mechanistic assumptions at the bumblebee colony level and allows to scale up population dynamics at the landscape level. The LandscapePhenoBee model simulates bee populations considering the impact of spatial heterogeneity and resource variability in time by combining a module for spatially explicit foraging with a model of population dynamics for an average colony in the landscape. The model was developed to be relatively simple with a few parameters, allowing the possibility to estimate the parameters given observed data on population growth, focal land use, and temperature if available. The model is parameterized to reproduce a theoretical representation of the bumblebee cycle colony pattern that has been previously observed and described (Becher et al., 2018; Benton, 2006; Crone & Williams, 2016; Duchateau & Velthuis, 1988; Goulson, 2003). To demonstrate the potential of the LandscapePhenoBee to describe the impact of climate extreme events, in this case drought, on pollination services and population dynamics in different types of landscapes, we mimicked drought events by simulating shortage in food resources in a floral phenology model part of LandscapePhenoBee. From model simulations, we found that the population size (the maximum number of workers produced at the peak of growth), population viability (queen daughters), and ecosystem services (pollination potential in flowering crops) increased with the amount of SNH in the landscape (Figure 4). The model simulations showed that the populations at the landscape level did not crash, nor did they reach the highest numbers all the time, showing that the model can capture variability in the output.

### 4.1 Sensitivity analysis

From the sensitivity analysis, we found that population dynamic parameters related to survival, growth, and the time for the start of queen production were the most influential. It is not surprising that the effect of 10% around the survival parameter can have a strong effect on the model results, but this was a result of treating all the parameters the same way for the sensitivity analysis (varying ±10% of their nominal value). All population parameters are considered constant during a major part of the season, except at the end, as the survival decreases and translate into increased mortality at the colony.

Further model development, allowing for changes in survival along the season, could be useful to explore different scenarios of worker mortality during colony growth. This would for example allow exploring threats to workers during their active time, such as effects of pesticides (Gill et al., 2012), parasites, or predation (Goulson et al., 2018), affecting their contribution to resource return to the colony and consequent development (Kerr et al., 2019).

### 4.2 SNH and nesting opportunities

SNH provide both nesting habitats and a continuous provision of food resources throughout the whole season. As per the design of our study, the number of nests increases with the amount of SNH. Therefore, the SNH determines the initial colony conditions for bumblebee population growth in the landscape. After that initial stage, flower resources will influence population growth. Estimating the location and the number of bumblebee nests in a landscape remains a challenge in pollination ecology, since nests cannot be easily surveyed (Knight et al., 2005). We did not simulate a specific bumblebee species, but a fictive common bumblebee that stands for several species including *B. terrestris, B. lucorum,* and *B. lapidarius*, and our initial conditions generated between 35 and 110 nests per potential nesting habitat area (corresponding to different nesting densities between 82 and 170 per km^2^). The density of nests varies a lot between landscapes, partly due to the amount of SNH, which makes it difficult to assess the differences between our results and those found in empirical studies, as well as how the nest densities are calculated. For example, Knight et al. (2005) estimated 29 for *B. terrestris* and 117/km^2^ for *B. lapidarius*, or 25 nests per km^2^ that Timberlake et al. (2021) estimated for *B. terrestris*.

### 4.3 Effects of SNH and impacts of drought

From the model outputs, the negative impact of drought was the highest in landscapes with a low proportion of SNH, affecting the constant food resources in the season. According to previous studies, SNH present an important role in maintaining bee populations in the presence of drought. While we are not aware of any published empirical studies of droughts on bumblebees in contrasting landscapes, the pattern observed in the model output is similar to what has been observed in assessing drought and short‐term temperature increases in taxa of flower‐visiting insects, since Oliver et al. (2013) showed that quantity and low degree of fragmentation of habitats reduce sensitivity to drought in butterflies. Oliver et al. (2013, 2015) also assessed recovery from drought but included many other factors involved, such as inter‐patch dynamics between generations, which are not directly comparable to our results. Papanikolaou et al. (2017) showed, using long‐term monitoring data from Germany, that a higher amount of SNH can mitigate the negative effects of short‐term increases in temperature on wild bee species richness. It is unclear whether the patterns found were due to the disproportionate importance of food resources in SNH under drought conditions, or whether these habitats provide cooler microhabitats. We did not consider differences in microclimates between habitats, which may be relevant in explaining interactive effects on bees between land‐use and climate extremes (as discussed in Papanikolaou et al., 2017).

Additionally, drought periods were implemented as a reduction in all floral resources in the landscape, and further model developments could consider drought resistance between different land‐use types, allowing for example to explore different drought‐resistant crop varieties.

### 4.4 Effects of MFC and impacts of drought

Our simulation results show that the impact of short periods of drought early in the season is not severe on population size but has negative impacts on both queen production and pollination potential. This can be explained as short drought periods have an intermediate decrease in food resources (Figures S2 and S3) and bees are able to compensate for the lack of food resources in space and time (maintaining the population size, i.e., number of workers). However, although population size can still reach a high number under drought conditions, the colonies may have missed the peak of early flowering or the peak is reduced, translating to a loss of resources available to produce queens.

The production of queens was higher in landscapes where there was a high enough proportion of late‐flowering crops. Where the ratio of early‐flowering crops to late‐flowering crops was too high, the production of queens decreased (Figure 4e).

In our model simulations, the more SNH, the more nests in the landscape, and the higher chance that a nest is close to floral resources in crops, which can explain the positive effect on pollination potential results. Pollination services from bees have been previously measured as visits per unit area (Lonsdorf et al., 2009), visits per flower (Rader et al., 2012), improved crop yield (Ricketts et al., 2016) or assumed as a direct proxy of species richness (Perennes et al., 2021). The pollination score used in this work approximates improved crop yield framed as pollination potential, describing how much crop flower resources can be benefited by bee visits and calculated based on visits per flower. Consequently, given the same number of foragers, when there are many flowers in the landscape, the crop flower become less efficiently pollinated. This can explain why we see from our model simulations that pollination potential is highly variable and, in the absence of drought, pollination potential decreases with an increasing proportion of early flowering crops (Figure 4f). On the other hand, in the presence of drought, the reduction of pollination services is steeper with increasing early MFC because that drought might have on the number of workers in the landscape (Figure 4d,f). The effects of drought in a plant‐pollinator interaction system affect both the crop and the pollinator, hence it is difficult to distinguish the drought effects on pollination. This is also the case in empirical studies. Plant crops under drought stress have reduced photosynthesis and thus decreased growth and produce lower reward and visual cues for pollinators (Descamps et al., 2020; Rering et al., 2020), affecting the nutritional quality and availability of floral resources for bees and having an impact on their survival (Wilson Rankin et al., 2020). Simultaneously, drought can induce changes in floral traits and morphology important for interactions with bumblebees (e.g., number of flowers, size of the flower, petal length and width, and depth of the nectar tube), and flowers might be seen as less attractive to forage on (or difficult to handle if they have a reduced size), affecting the bumblebee behavior and resulting in a reduction in the flower visits, as experimental setups found (Höfer et al., 2021; Kuppler et al., 2021).

### 4.5 The use of temperature sums in the model

GDD is the accumulation of temperature above a certain base temperature for each calendar day, making it a good indicator to account for both spatial and temporal variation in temperature, including the lower developmental threshold in which plant growth development and flowering are possible. Since bees are sensitive to temperature change (Martinet et al., 2020; Pawlikowski et al., 2020), GDD has a great potential to predict insect phenology, that is, the development of insects (Cayton et al., 2015).

In this study, we have used a theoretical generalized seasonal progression, to represent a theoretical GDD value to mark the start and end of flowering for the flowering period, spring bee emergence, and worker foraging. A choice of the model was to also use an arbitrary GDD to trigger the initiation of the production of daughter queens. While the emergence of spring queens is triggered by temperature (Alford, 1969; Goodwin, 1995), the production of new queens is a complex combination of factors involving resources in the landscape, temperature, and health of the colony (Goulson, 2003). There are several approaches to model the switch point including time (Crone & Williams, 2016), and assessing the daily ratio of larvae‐worker below a certain threshold (e.g., 3 used in BumblebeeHAVE (Becher et al., 2018)). By adding GDD in our model as a switching point, we could add variability to the time of queen production, by shifting the day of production, instead of a fixed day in time as in Crone and Williams (2016). We acknowledge that using temperature as a unique switching condition to produce new queens is not perfect, and some studies do not find temperature as the main reason for switching point (Holland & Bourke, 2015; Vogt, 1986). However, there is a lack of experimental studies that control for different temperatures, or field data and modeling to construct a better understanding of how bumblebees or other social insect pollinators respond to changes in temperatures (but see Zaragoza‐Trello et al., 2021). Additional carefully controlled experimental studies, for example with variable temperature regimes, in combination with data from the field and modeling, should help construct a fuller understanding of how major social insect pollinators are likely to respond to climate change.

An additional aspect worth mentioning is that we did not consider in our model the production of males, and the complex colony dynamics of queen‐worker conflict derived from the switching point of producing reproductives in the colony (Goulson, 2003). Given the nature of the model to produce a representation of the bumblebee colony cycle pattern, the number of daughter queens was slightly higher in landscapes that had more late‐flowering crops, as shown in empirical studies (Rundlöf et al., 2014). Results from the simulation indicate that landscapes with more late‐flowering crops would increase the number of workers, and therefore bring more resources to the colony and produce more queens.

## 5 CONCLUSIONS

Climate and land‐use changes are two drivers of bee population decline that should be considered in combination. The LandscapePhenoBee is a new mechanistic pollination model that can account for landscape heterogeneity and temporal variability in resources to bees yet keeping the model relatively simple with a few parameters. By introducing climate‐induced temporal variability of food resources, this model contributes to the methodology of studying and predicting the impact of important drivers and extreme events on wild bees. As an example, the theoretical model shows the ability to qualitatively reproduce patterns observed in the very limited number of studies combining landscape‐scale availability of resources and drought (Oliver et al., 2013, 2015; Papanikolaou et al., 2017).

## AUTHOR CONTRIBUTIONS

**Maria Blasi:** Conceptualization (equal); formal analysis (equal); funding acquisition (supporting); methodology (equal); resources (supporting); visualization (lead); writing – original draft (lead); writing – review and editing (lead). **Yann Clough:** Conceptualization (equal); funding acquisition (supporting); writing – original draft (supporting); writing – review and editing (equal). **Anna Maria Jönsson:** Conceptualization (supporting); writing – original draft (supporting); writing – review and editing (equal). **Ullrika Sahlin:** Conceptualization (equal); formal analysis (equal); funding acquisition (supporting); investigation (equal); methodology (lead); writing – original draft (lead); writing – review and editing (equal).

## CONFLICT OF INTEREST

The authors declare no conflict of interest.

## Supporting information


Appendix S1

## Data Availability

The code and simulated data that support the findings of this study are openly available in the GitHub repository link: https://github.com/mblasirom/LandscapePhenoBee and Dryad https://doi.org/10.5061/dryad.pc866t1rj.
