# Supplementary material for: A model of wild bee populations accounting for spatial heterogeneity and climate‐induced temporal variability of food resources at the landscape level
Source: Ecol Evol. 2022 Jun 17;12(6):e9014. doi: 10.1002/ece3.9014 (PMC9205664; doi:10.1002/ece3.9014)
Supplement: Supplementary file 1 — Appendix S1 [file ECE3-12-e9014-s001.docx]

**Appendix for:**

**A model of wild bee populations accounting for spatial heterogeneity and climate-induced temporal variability of food resources at the landscape level**


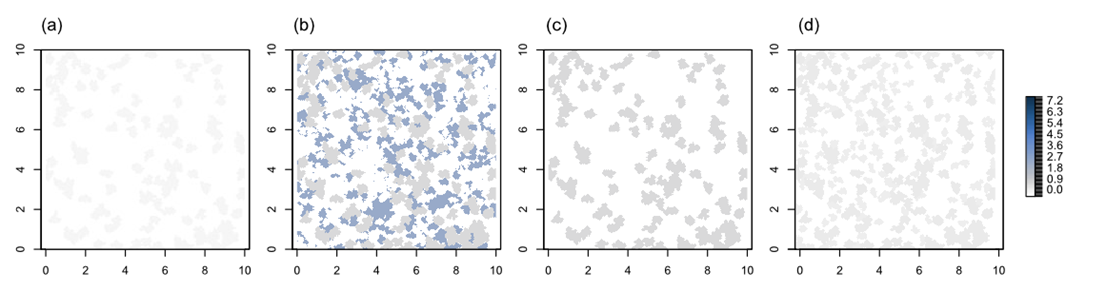


Figure S1. Temporal distribution of resources during four different weeks (out of the 34 weeks considered in the study). The patches in each graph represent land-use covers and the colour gradient represents the availability of floral resources at a given time. (a) Represents the beginning of the season with only some floral resources available, from semi-natural habitats. (b) The peak of floral resources during the flowering of the early crop, and semi-natural habitats. (c) After the peak of the early flowering crop, only floral resources from semi-natural habitats are available. (d) At the end of the season, representing the peak of blooming of late flowering crop, which provides fewer floral resources than the peak of early flowering crop, shown in (b). The scale values indicate a fictive amount of resources present at the landscape for a given time. The darker the color, the more resources.


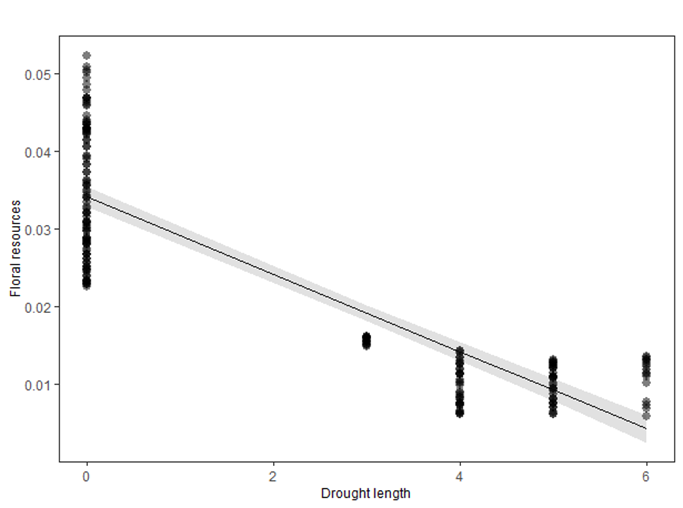


Figure S2. Floral resources (area under the curve for the total floral resources over the season) were severely affected by different drought durations (p-value < 0.001).


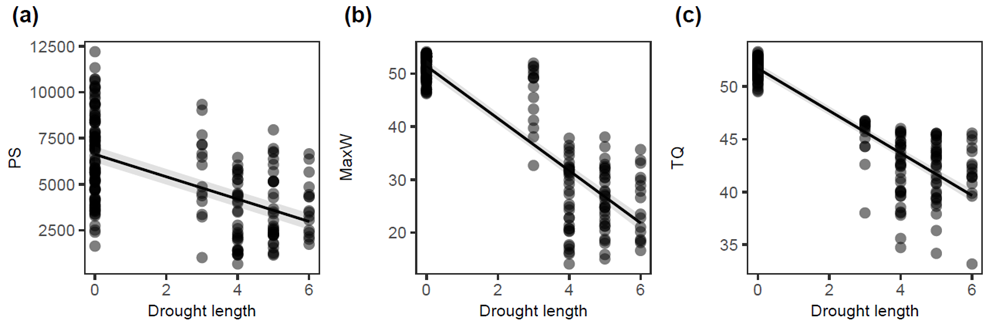


Figure S3. Effect of drought duration on the three response variables pollination potential (a), maximum production of workers (b), and the number of daughter queens per nest (c).


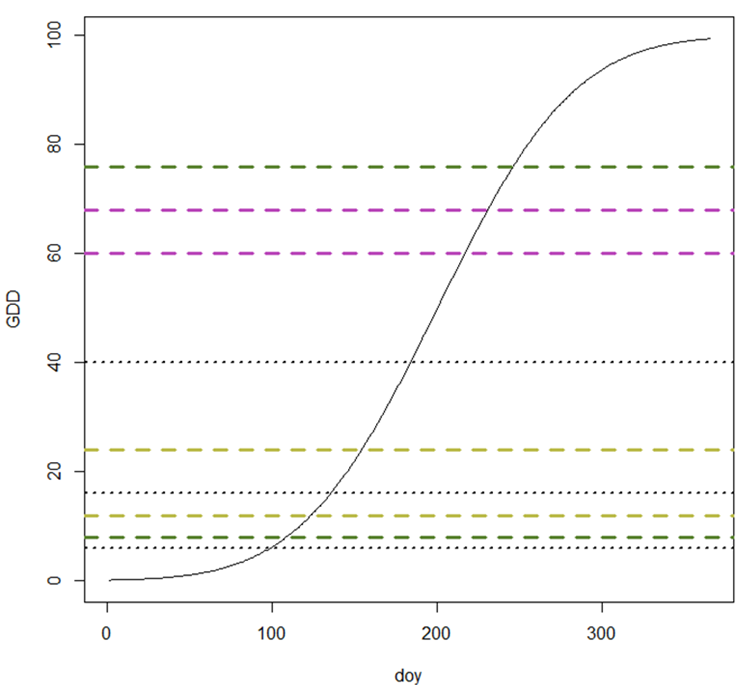


Figure S4. Representation of the generalised seasonal progression with the accumulation of arbitrary growing degree days (GDD) according to the day of the year (doy). Color lines indicate the start and end of the flowering season for each land cover used in this paper: green for semi-natural habitats, yellow for early crop, and purple for late crop. Black horizontal dotted lines represent (in chronological order) the emergence of spring queens (φ), the start of workers foraging (µ), and the production of daughter queens (ψ).


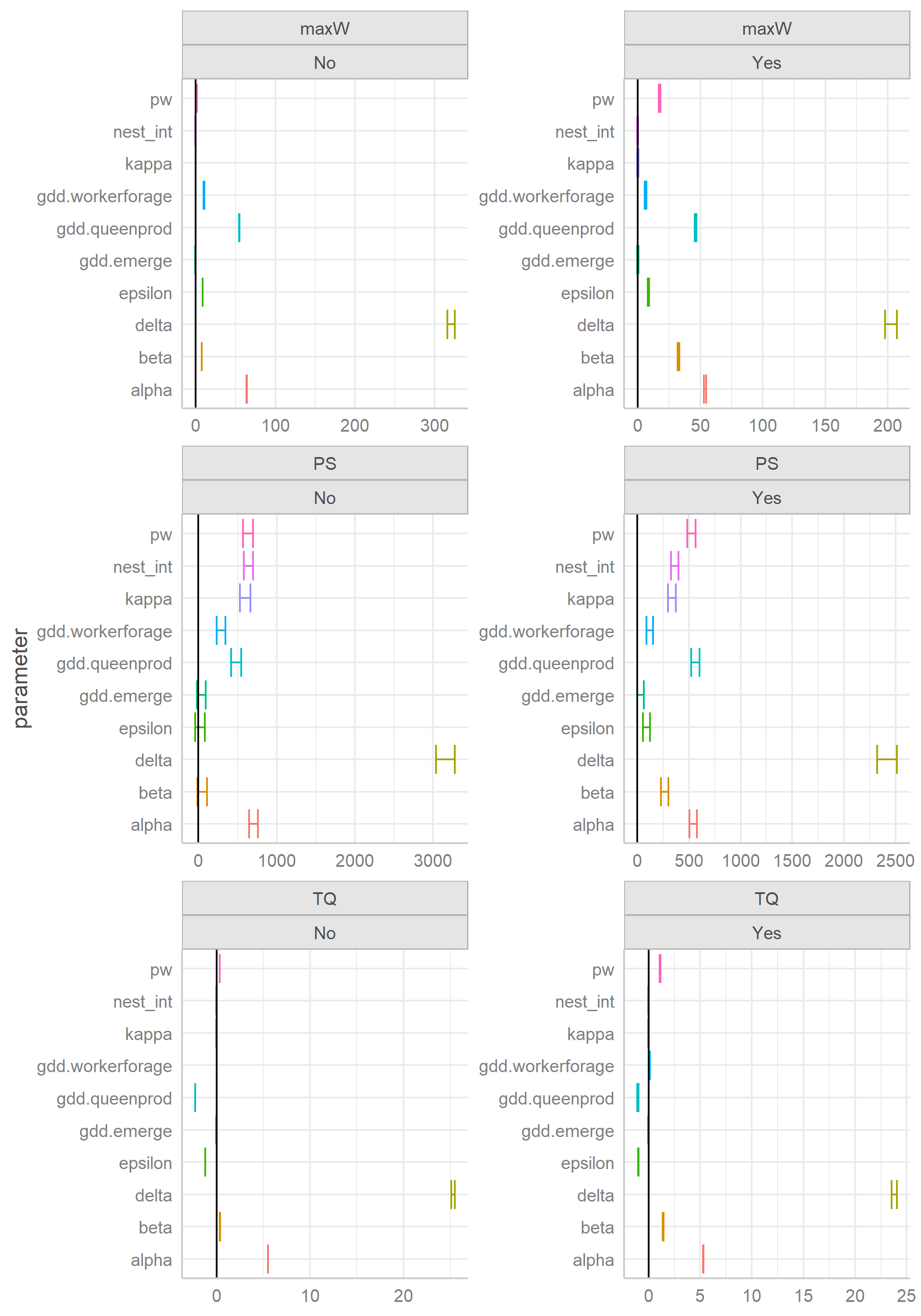


Figure S5. Sensitivity analysis. Representation of the parameter influence in the effect of each response variable estimates: maxW (number of workers), PS (pollination potential), and TQ (number of queens). Drought effects are the panels shown as Yes (presence of drought) and No (absence of drought). The error bars indicate the range of the estimates. See table S4 for the parameter influence in the effect of each independent variable estimate (% early MFC, % SNH and Drought).


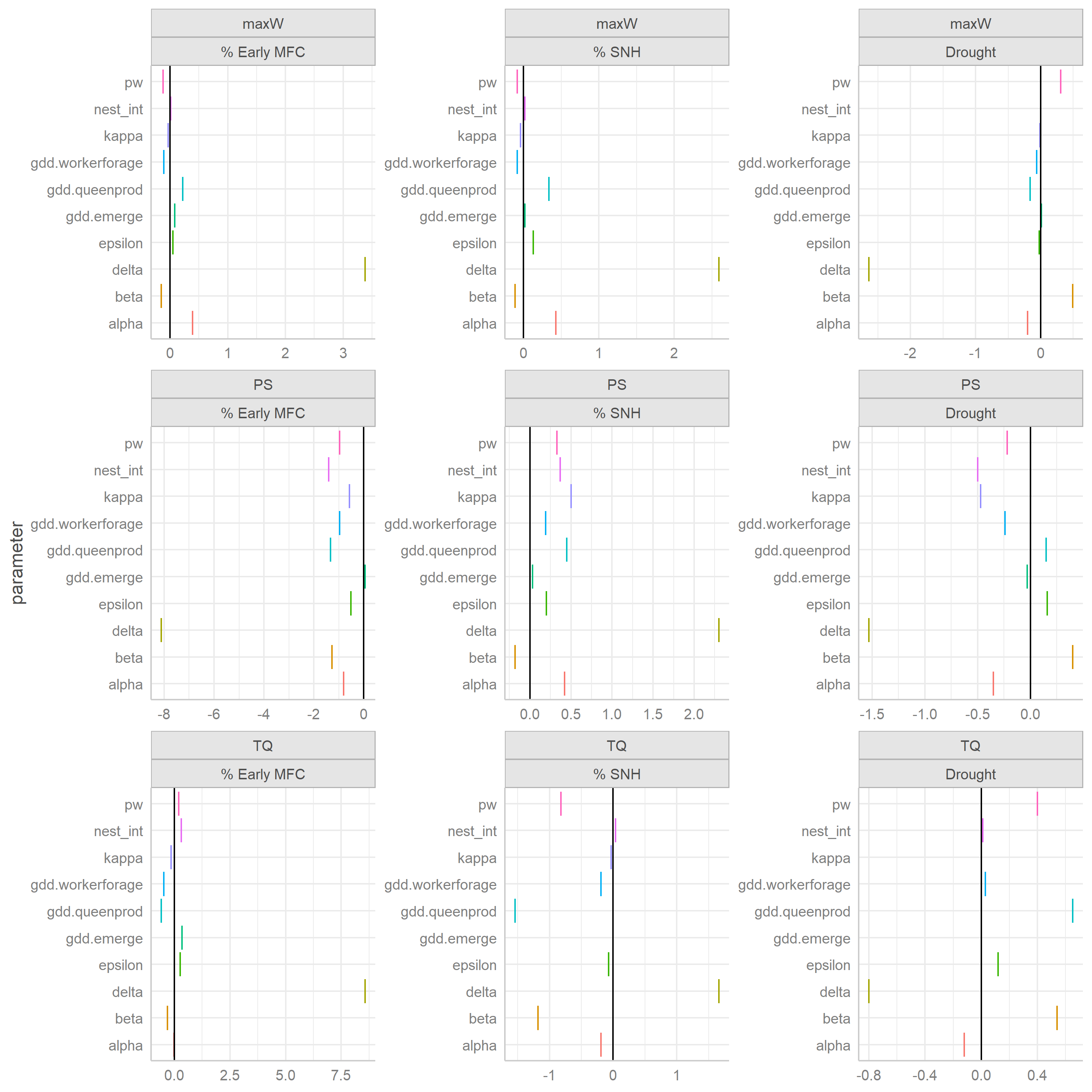


Figure S6. Sensitivity analysis. Representation of the parameter influence in the effect of each independent variable estimate (% early MFC, % SNH and Drought), for each response variable maxW (number of workers), PS (pollination potential), and TQ (number of queens).


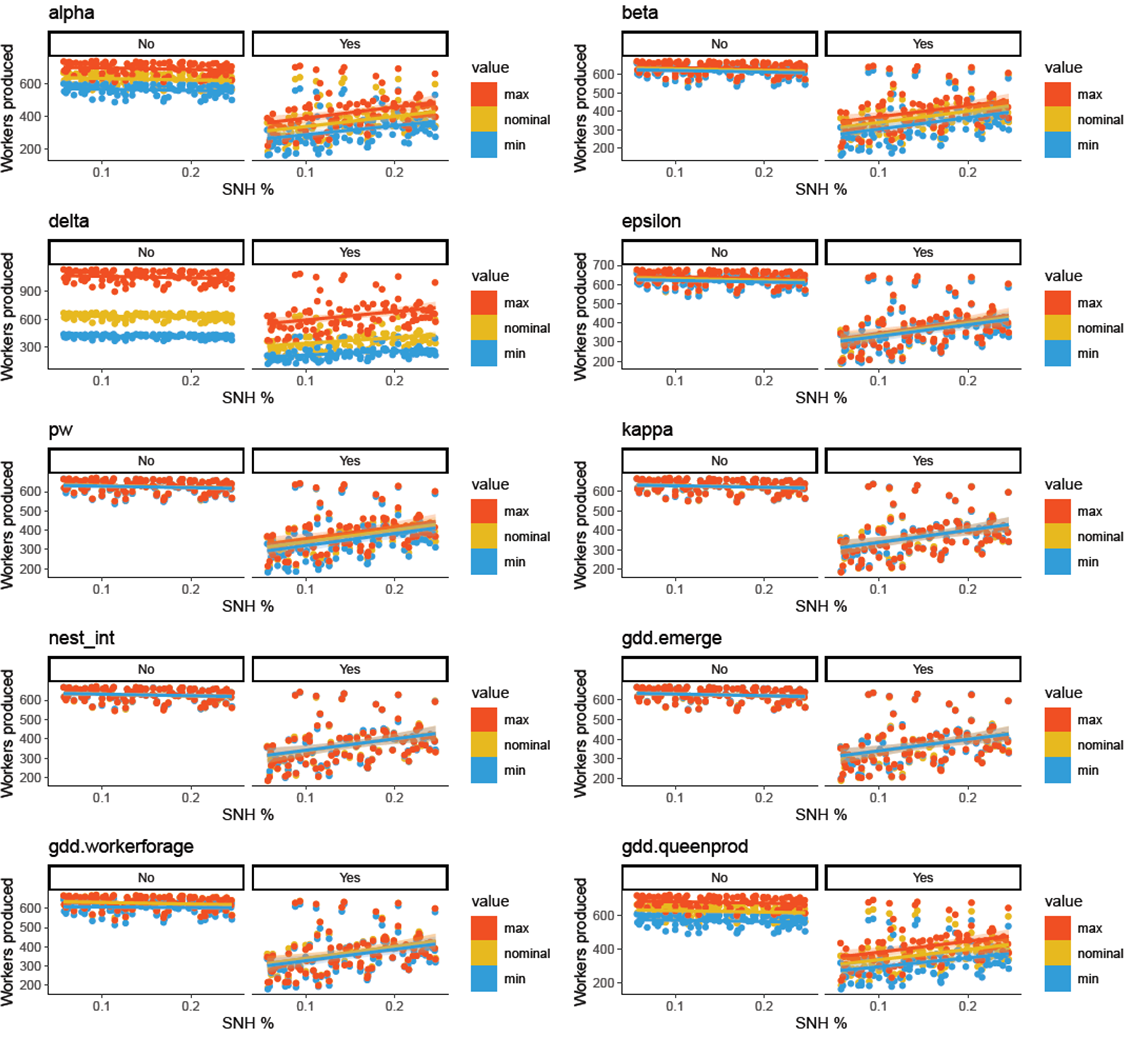


Figure S7. Evaluation of the parameter sensitivity for the production of workers. Drought effects are represented as Yes (presence of drought) and No (absence of drought).


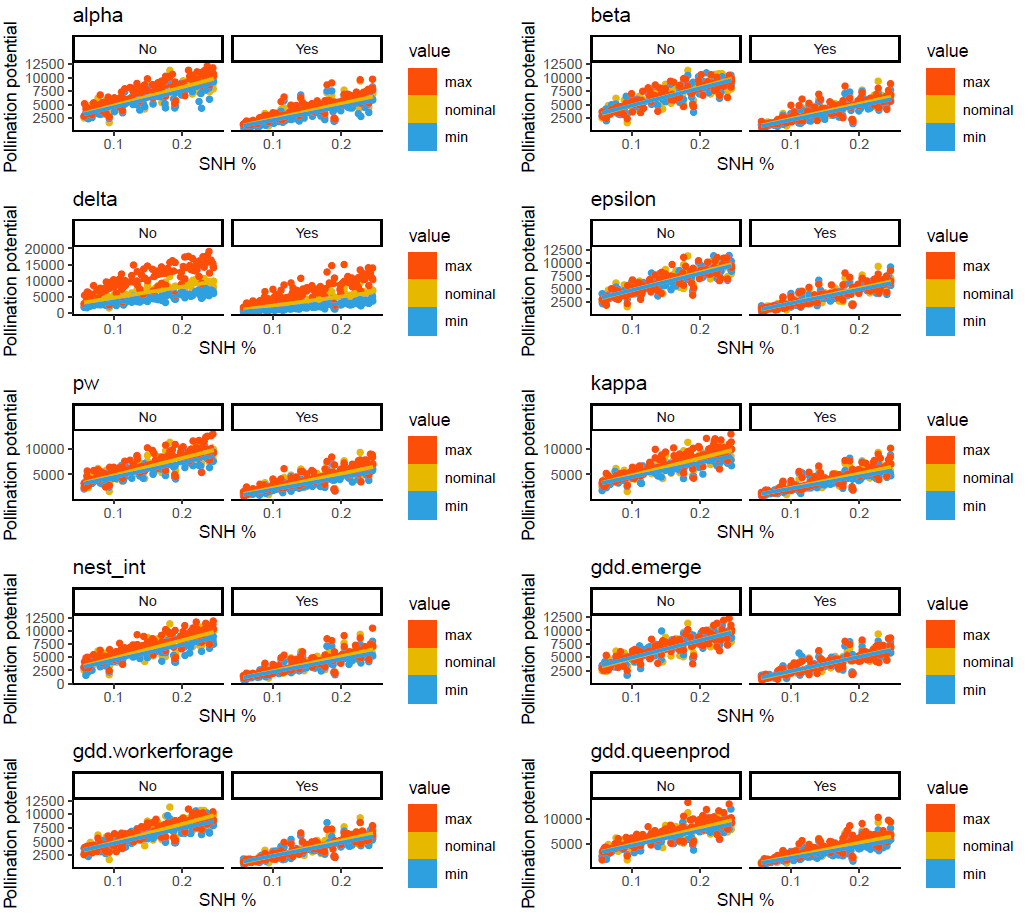
Figure S8. Evaluation of the parameter sensitivity for the pollination potential. Drought effects are represented as Yes (presence of drought) and No (absence of drought).


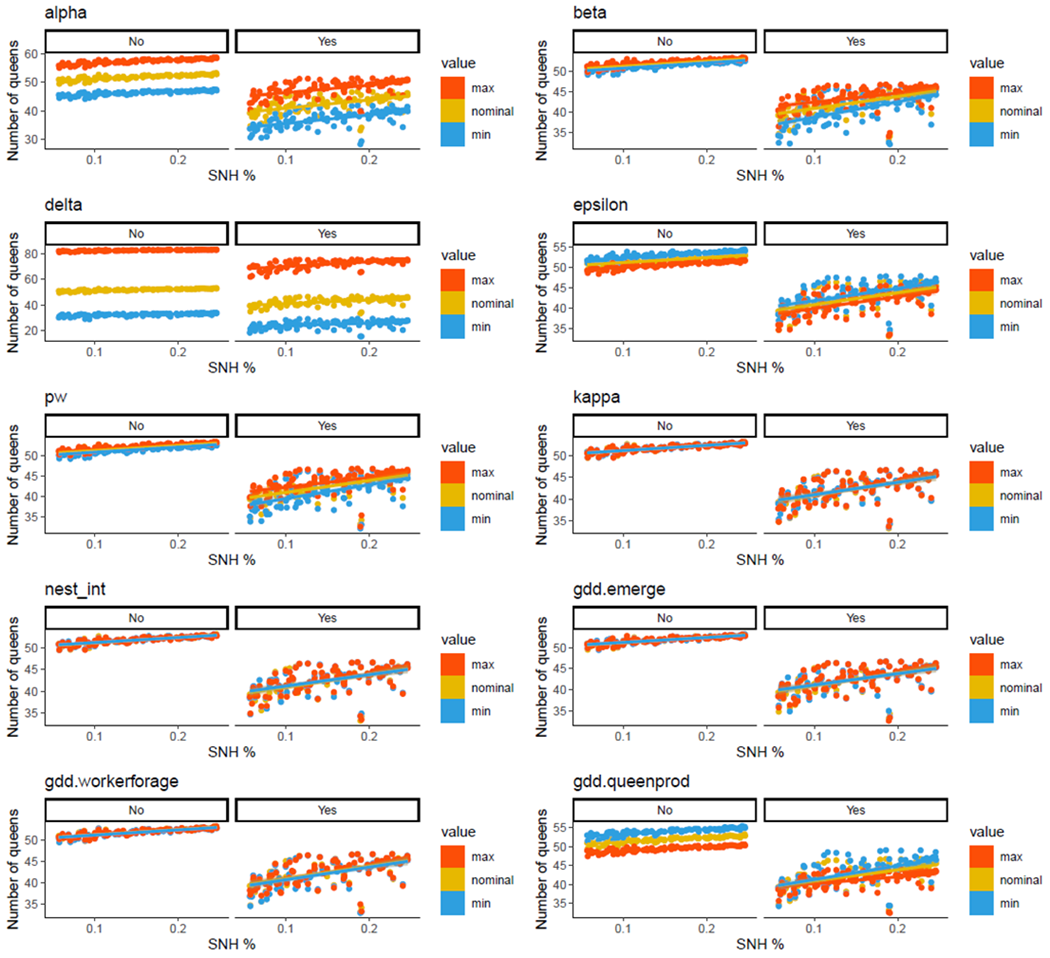


Figure S9. Evaluation of the parameter sensitivity for the total number of queens. Drought effects are represented as Yes (presence of drought) and No (absence of drought).

Table S1. Comparison between Häussler et al 2017 and LandscapePhenoBee, presented in this paper.

|  | **Häussler et al. 2017** | **LandscapePhenoBee** |
| --- | --- | --- |
| Aim of the model | Effect of landscape on flower visitation rate, long term effects | Effect of landscape and climate on colony dynamics at the landscape level within a season (short term effects) |
| Model outputs | Col no., survival, flower visitation rates | Average number of workers produced per season, number of daughter queens, pollinator potential |
| Number of floral periods distinguished within a season (year) | Two periods (spring and summer) | Weekly (n. weeks) |
| Colony dynamics | Queens foraging (period 1), workers foraging and new queens are produced (period 2) | Spring queens foraging, workers foraging, new queens produced |
| Climate components | Not considered | Vegetation development and bee colony development is dependent of a theoretical generalized seasonal progression, that can be substituted by real GDD values. |
| Floral resources | Floral resources are fixed for period 1 and period 2 | Floral resources change every week according to the theoretical generalized seasonal progression- interpreted as a arbitrary GDD. |
| Years | 35 years (long-term) | 1 year |

| Parameter name | Description | Nominal value | Units |
| --- | --- | --- | --- |
| α | Maximum number of workers produced during stages A2 to B2 | 10 |  |
| β | For which amount of resources half of the potential number of workers is being produced during stages A2 to B2 | 1 |  |
| δ | Weekly survival rate of workers and daughter queens during stages A2 to B2 | 0.9 |  |
| ε | Proportion of colony workers among newly produced individuals in the nest | 0.2 |  |
| κ | In the pollination score, is the parameter adjusting how quickly the visitation rate per floral resource reaches the maximum pollination | 1 |  |
| pw | The proportion of workers foraging used to calculate the total number of visitation rate when workers are foraging | 0.8 |  |
| Nest density | Density of nests in the semi-natural habitats | 0.01 |  |
| φ | temperature sum for queen emergence | 6 | GDD (+5 °C) * |
| µ | temperature sum for the start of workers foraging | 16 | GDD (+5 °C) * |
| ψ | temperature sum for the start of production of daughter queens | 40 | GDD (+5 °C) * |

Table S2. Population model parameters. Nominal value of the parameters is chosen accordingly to produce a realistic pattern of the bumblebee colony dynamics.

*arbitrary GDD values for the theoretical generalized seasonal progression.

| Output name | Description |
| --- | --- |
| MaxW | Maximum number of foragers per nest per week when peak of growth |
| TQ | Total number of daughter queens produced per nest |
| F | Sum of the floral values in the landscape per m2 |
| PS | Pollination potential |

Table S3. Model outputs for population dynamics and pollination services results.

Table S4. Sensitivity analysis results. Coefficient of change (S) are shown from the regression analysis to evaluate the effect of % SNH, % early MFC and Drought on the maximum number of workers (MaxW), the total number of queens (TQ), and the pollination potential (PS).

| **Response variable** | **Parameter** | **% SNH** | **% early MFC** | **Drought** |
| --- | --- | --- | --- | --- |
| Maximum number workers (MaxW) | $\alpha_{t}$ | 0.43 | 0.39 | -0.20 |
|  | $\beta_{t}$ | -0.11 | -0.15 | 0.49 |
|  | $\delta_{t}$ | 2.59 | 3.38 | -2.64 |
|  | ε | 0.13 | 0.05 | -0.02 |
|  | κ | -0.04 | -0.03 | -0.01 |
|  | pw | -0.08 | -0.12 | 0.31 |
|  | Nest density | 0.02 | 0.01 | 0.00 |
|  | φ | 0.02 | 0.08 | 0.01 |
|  | µ | -0.08 | -0.11 | -0.06 |
|  | ψ | 0.34 | 0.22 | -0.16 |
| Number of queens (TQ) | $\alpha_{t}$ | -0.19 | -0.01 | -0.12 |
|  | $\beta_{t}$ | -1.18 | -0.30 | 0.54 |
|  | $\delta_{t}$ | 1.66 | 8.57 | -0.80 |
|  | ε | -0.07 | 0.26 | 0.12 |
|  | κ | -0.03 | -0.14 | 0.00 |
|  | pw | -0.82 | 0.20 | 0.40 |
|  | Nest density | 0.04 | 0.32 | 0.01 |
|  | φ | 0.00 | 0.35 | 0.00 |
|  | µ | -0.19 | -0.47 | 0.03 |
|  | ψ | -1.54 | -0.58 | 0.65 |
| Pollination potential (PS) | $\alpha_{t}$ | 0.42 | -0.81 | -0.35 |
|  | $\beta_{t}$ | -0.18 | -1.27 | 0.40 |
|  | $\delta_{t}$ | 2.30 | -8.11 | -1.53 |
|  | ε | 0.20 | -0.51 | 0.16 |
|  | κ | 0.50 | -0.57 | -0.47 |
|  | pw | 0.33 | -0.97 | -0.22 |
|  | Nest density | 0.37 | -1.40 | -0.50 |
|  | φ | 0.03 | 0.05 | -0.03 |
|  | µ | 0.19 | -0.97 | -0.24 |
|  | ψ | 0.45 | -1.34 | 0.15 |

Table S5. Regression analysis used for the sensitivity analysis to evaluate the effect of % SNH, % early MFC and Drought on the maximum number of workers (MaxW). Model estimates, standard error (Std. error), and standardized regression coefficient (t-value) are provided for each regression analysis. Values for max and min correspond to the model results with that specific parameter value modified +10% (max), or -10% (min).

| **Model** | **Parameter** | **Value** | **Predictors** | **Estimates** | **std. Error** | **Statistic** |
| --- | --- | --- | --- | --- | --- | --- |
| mod1 | reference | reference | (Intercept) | 449.47 | 18.08 | 24.86 |
|  | reference | reference | % SNH | 524.42 | 78.21 | 6.71 |
|  | reference | reference | % early MFC | 400.45 | 37.63 | 10.64 |
|  | reference | reference | Drought | -253.67 | 8.75 | -28.98 |
| mod2 | α | max | (Intercept) | 498.45 | 19.20 | 25.97 |
|  | α | max | % SNH | 568.41 | 83.03 | 6.85 |
|  | α | max | % early MFC | 434.55 | 39.95 | 10.88 |
|  | α | max | Drought | -263.56 | 9.29 | -28.36 |
| mod3 | α | min | (Intercept) | 397.63 | 16.51 | 24.08 |
|  | α | min | % SNH | 481.25 | 71.42 | 6.74 |
|  | α | min | % early MFC | 376.20 | 34.36 | 10.95 |
|  | α | min | Drought | -242.92 | 7.99 | -30.39 |
| mod4 | β | max | (Intercept) | 460.63 | 17.04 | 27.03 |
|  | β | max | % SNH | 516.68 | 73.72 | 7.01 |
|  | β | max | % early MFC | 388.98 | 35.47 | 10.97 |
|  | β | max | Drought | -230.58 | 8.25 | -27.94 |
| mod5 | β | min | (Intercept) | 435.09 | 18.94 | 22.97 |
|  | β | min | % SNH | 541.65 | 81.91 | 6.61 |
|  | β | min | % early MFC | 413.93 | 39.41 | 10.50 |
|  | β | min | Drought | -280.10 | 9.17 | -30.55 |
| mod6 | δ | max | (Intercept) | 750.84 | 28.92 | 25.96 |
|  | δ | max | % SNH | 841.12 | 125.11 | 6.72 |
|  | δ | max | % early MFC | 724.61 | 60.19 | 12.04 |
|  | δ | max | Drought | -413.32 | 14.00 | -29.52 |
| mod7 | δ | min | (Intercept) | 298.98 | 12.56 | 23.80 |
|  | δ | min | % SNH | 354.61 | 54.33 | 6.53 |
|  | δ | min | % early MFC | 249.61 | 26.14 | 9.55 |
|  | δ | min | Drought | -177.71 | 6.08 | -29.22 |
| mod8 | ε | max | (Intercept) | 453.56 | 18.16 | 24.98 |
|  | ε | max | % SNH | 543.08 | 78.53 | 6.92 |
|  | ε | max | % early MFC | 408.02 | 37.79 | 10.80 |
|  | ε | max | Drought | -254.70 | 8.79 | -28.98 |
| mod9 | ε | min | (Intercept) | 439.96 | 17.91 | 24.57 |
|  | ε | min | % SNH | 520.40 | 77.46 | 6.72 |
|  | ε | min | % early MFC | 403.52 | 37.27 | 10.83 |
|  | ε | min | Drought | -253.42 | 8.67 | -29.23 |
| mod10 | pw | max | (Intercept) | 453.42 | 17.38 | 26.10 |
|  | pw | max | % SNH | 521.88 | 75.15 | 6.94 |
|  | pw | max | % early MFC | 391.17 | 36.16 | 10.82 |
|  | pw | max | Drought | -238.73 | 8.41 | -28.38 |
| mod11 | pw | min | (Intercept) | 442.27 | 18.72 | 23.62 |
|  | pw | min | % SNH | 543.23 | 80.98 | 6.71 |
|  | pw | min | % early MFC | 411.07 | 38.96 | 10.55 |
|  | pw | min | Drought | -270.51 | 9.06 | -29.84 |
| mod12 | κ | max | (Intercept) | 450.00 | 18.18 | 24.75 |
|  | κ | max | % SNH | 525.26 | 78.63 | 6.68 |
|  | κ | max | % early MFC | 396.94 | 37.83 | 10.49 |
|  | κ | max | Drought | -254.37 | 8.80 | -28.90 |
| mod13 | κ | min | (Intercept) | 446.87 | 18.07 | 24.73 |
|  | κ | min | % SNH | 536.71 | 78.16 | 6.87 |
|  | κ | min | % early MFC | 401.23 | 37.61 | 10.67 |
|  | κ | min | Drought | -254.15 | 8.75 | -29.05 |
| mod14 | nest_int | max | (Intercept) | 450.36 | 18.01 | 25.00 |
|  | nest_int | max | % SNH | 521.31 | 77.92 | 6.69 |
|  | nest_int | max | % early MFC | 400.14 | 37.49 | 10.67 |
|  | nest_int | max | Drought | -253.39 | 8.72 | -29.05 |
| mod15 | nest_int | min | (Intercept) | 452.42 | 17.80 | 25.41 |
|  | nest_int | min | % SNH | 512.65 | 77.00 | 6.66 |
|  | nest_int | min | % early MFC | 396.67 | 37.05 | 10.71 |
|  | nest_int | min | Drought | -253.21 | 8.62 | -29.38 |
| mod16 | φ | max | (Intercept) | 448.64 | 18.01 | 24.92 |
|  | φ | max | % SNH | 518.19 | 77.88 | 6.65 |
|  | φ | max | % early MFC | 406.26 | 37.47 | 10.84 |
|  | φ | max | Drought | -253.30 | 8.72 | -29.06 |
| mod17 | φ | min | (Intercept) | 453.82 | 18.04 | 25.16 |
|  | φ | min | % SNH | 507.18 | 78.03 | 6.50 |
|  | φ | min | % early MFC | 393.88 | 37.54 | 10.49 |
|  | φ | min | Drought | -254.00 | 8.73 | -29.08 |
| mod18 | μ | max | (Intercept) | 448.77 | 17.97 | 24.98 |
|  | μ | max | % SNH | 528.10 | 77.71 | 6.80 |
|  | μ | max | % early MFC | 401.17 | 37.39 | 10.73 |
|  | μ | max | Drought | -254.32 | 8.70 | -29.24 |
| mod19 | μ | min | (Intercept) | 417.46 | 17.08 | 24.44 |
|  | μ | min | % SNH | 556.60 | 73.88 | 7.53 |
|  | μ | min | % early MFC | 426.21 | 35.55 | 11.99 |
|  | μ | min | Drought | -245.79 | 8.27 | -29.72 |
| mod20 | ψ | max | (Intercept) | 494.04 | 18.66 | 26.48 |
|  | ψ | max | % SNH | 553.43 | 80.71 | 6.86 |
|  | ψ | max | % early MFC | 419.24 | 38.83 | 10.80 |
|  | ψ | max | Drought | -260.27 | 9.03 | -28.81 |
| mod21 | ψ | min | (Intercept) | 403.62 | 16.82 | 24.00 |
|  | ψ | min | % SNH | 478.59 | 72.75 | 6.58 |
|  | ψ | min | % early MFC | 386.58 | 35.00 | 11.04 |
|  | ψ | min | Drought | -243.29 | 8.14 | -29.88 |

Table S6. Regression analysis used for the sensitivity analysis to evaluate the effect of % SNH, % early MFC, and Drought on the pollination potential. Model estimates, standard error (Std. error), and standardized regression coefficient (t-value) are provided for each regression analysis. Values for max and min correspond to the model results with that specific parameter value modified +10% (max), or -10% (min).

| **Model** | **Parameter** | **Value** | **Predictors** | **Estimates** | **std. Error** | **Statistic** |
| --- | --- | --- | --- | --- | --- | --- |
| mod1 | reference | reference | (Intercept) | 2632.26 | 388.95 | 6.77 |
|  | reference | reference | % SNH | 30352.93 | 1718.37 | 17.66 |
|  | reference | reference | % early MFC | -2705.94 | 826.80 | -3.27 |
|  | reference | reference | Drought | -2748.76 | 103.70 | -26.51 |
| mod2 | α | max | (Intercept) | 3376.86 | 402.90 | 8.38 |
|  | α | max | % SNH | 31835.73 | 1780.17 | 17.88 |
|  | α | max | % early MFC | -3083.33 | 856.53 | -3.60 |
|  | α | max | Drought | -2984.38 | 106.87 | -27.93 |
| mod3 | α | min | (Intercept) | 2665.75 | 415.67 | 6.41 |
|  | α | min | % SNH | 25840.98 | 1840.29 | 14.04 |
|  | α | min | % early MFC | -2168.77 | 885.46 | -2.45 |
|  | α | min | Drought | -2659.08 | 97.00 | -27.41 |
| mod4 | β | max | (Intercept) | 3113.08 | 431.58 | 7.21 |
|  | β | max | % SNH | 28009.13 | 1912.80 | 14.64 |
|  | β | max | % early MFC | -3106.68 | 920.35 | -3.38 |
|  | β | max | Drought | -2536.65 | 92.47 | -27.43 |
| mod5 | β | min | (Intercept) | 2487.98 | 385.90 | 6.45 |
|  | β | min | % SNH | 28948.80 | 1702.71 | 17.00 |
|  | β | min | % early MFC | -1499.53 | 819.26 | -1.83 |
|  | β | min | Drought | -2971.83 | 109.88 | -27.05 |
| mod6 | δ | max | (Intercept) | 5788.29 | 747.52 | 7.74 |
|  | δ | max | % SNH | 45669.84 | 3311.05 | 13.79 |
|  | δ | max | % early MFC | -8302.04 | 1593.11 | -5.21 |
|  | δ | max | Drought | -3748.54 | 168.47 | -22.25 |
| mod7 | δ | min | (Intercept) | 1634.81 | 231.11 | 7.07 |
|  | δ | min | % SNH | 19878.41 | 1016.22 | 19.56 |
|  | δ | min | % early MFC | -919.88 | 488.95 | -1.88 |
|  | δ | min | Drought | -2260.18 | 76.00 | -29.74 |
| mod8 | ε | max | (Intercept) | 2305.72 | 430.16 | 5.36 |
|  | ε | max | % SNH | 31568.73 | 1904.29 | 16.58 |
|  | ε | max | % early MFC | -2437.17 | 916.25 | -2.66 |
|  | ε | max | Drought | -2631.21 | 101.05 | -26.04 |
| mod9 | ε | min | (Intercept) | 2390.83 | 371.54 | 6.43 |
|  | ε | min | % SNH | 29132.36 | 1641.00 | 17.75 |
|  | ε | min | % early MFC | -1392.05 | 789.57 | -1.76 |
|  | ε | min | Drought | -2774.29 | 100.55 | -27.59 |
| mod10 | pw | max | (Intercept) | 3170.76 | 464.57 | 6.83 |
|  | pw | max | % SNH | 31335.23 | 2057.27 | 15.23 |
|  | pw | max | % early MFC | -2868.96 | 989.86 | -2.90 |
|  | pw | max | Drought | -2878.17 | 106.64 | -26.99 |
| mod11 | pw | min | (Intercept) | 2329.76 | 331.32 | 7.03 |
|  | pw | min | % SNH | 26397.88 | 1461.53 | 18.06 |
|  | pw | min | % early MFC | -1504.34 | 703.22 | -2.14 |
|  | pw | min | Drought | -2656.83 | 95.45 | -27.83 |
| mod12 | κ | max | (Intercept) | 2857.93 | 444.70 | 6.43 |
|  | κ | max | % SNH | 32513.62 | 1966.64 | 16.53 |
|  | κ | max | % early MFC | -2599.67 | 946.25 | -2.75 |
|  | κ | max | Drought | -2992.99 | 111.83 | -26.76 |
| mod13 | κ | min | (Intercept) | 2457.82 | 384.18 | 6.40 |
|  | κ | min | % SNH | 25784.36 | 1702.13 | 15.15 |
|  | κ | min | % early MFC | -1614.25 | 818.98 | -1.97 |
|  | κ | min | Drought | -2470.57 | 84.84 | -29.12 |
| mod14 | nest_int | max | (Intercept) | 3171.23 | 410.06 | 7.73 |
|  | nest_int | max | % SNH | 31471.83 | 1812.86 | 17.36 |
|  | nest_int | max | % early MFC | -3054.90 | 872.26 | -3.50 |
|  | nest_int | max | Drought | -3011.52 | 105.24 | -28.62 |
| mod15 | nest_int | min | (Intercept) | 2277.91 | 364.23 | 6.25 |
|  | nest_int | min | % SNH | 26058.68 | 1611.53 | 16.17 |
|  | nest_int | min | % early MFC | -1201.19 | 775.39 | -1.55 |
|  | nest_int | min | Drought | -2464.47 | 88.93 | -27.71 |
| mod16 | φ | max | (Intercept) | 2746.01 | 419.81 | 6.54 |
|  | φ | max | % SNH | 29843.23 | 1857.84 | 16.06 |
|  | φ | max | % early MFC | -2416.30 | 893.90 | -2.70 |
|  | φ | max | Drought | -2786.13 | 100.96 | -27.60 |
| mod17 | φ | min | (Intercept) | 2794.49 | 366.34 | 7.63 |
|  | φ | min | % SNH | 28751.77 | 1617.52 | 17.78 |
|  | φ | min | % early MFC | -2221.38 | 778.27 | -2.85 |
|  | φ | min | Drought | -2775.35 | 100.88 | -27.51 |
| mod18 | μ | max | (Intercept) | 2697.21 | 341.36 | 7.90 |
|  | μ | max | % SNH | 29969.59 | 1504.67 | 19.92 |
|  | μ | max | % early MFC | -2482.77 | 723.98 | -3.43 |
|  | μ | max | Drought | -2795.46 | 101.81 | -27.46 |
| mod19 | μ | min | (Intercept) | 2279.16 | 385.61 | 5.91 |
|  | μ | min | % SNH | 26243.70 | 1708.48 | 15.36 |
|  | μ | min | % early MFC | -761.79 | 822.04 | -0.93 |
|  | μ | min | Drought | -2460.87 | 85.11 | -28.91 |
| mod20 | ψ | max | (Intercept) | 3061.33 | 451.74 | 6.78 |
|  | ψ | max | % SNH | 32207.77 | 2001.07 | 16.10 |
|  | ψ | max | % early MFC | -3442.66 | 962.82 | -3.58 |
|  | ψ | max | Drought | -2668.49 | 101.22 | -26.36 |
| mod21 | ψ | min | (Intercept) | 2706.31 | 363.22 | 7.45 |
|  | ψ | min | % SNH | 26042.11 | 1604.18 | 16.23 |
|  | ψ | min | % early MFC | -2044.47 | 771.85 | -2.65 |
|  | ψ | min | Drought | -2831.06 | 98.58 | -28.72 |

Table S7. Sensitivity analysis results. Regression analysis to evaluate the effect of % SNH, % early MFC and Drought on the number of daughter queens. Model estimates, standard error (Std. error), and standardized regression coefficient (t-value) are provided for each regression analysis. Values for max and min correspond to the model results with that specific parameter value modified +10% (max), or -10% (min).

| **Model** | **Parameter** | **Value** | **Predictors** | **Estimates** | **std. Error** | **Statistic** |
| --- | --- | --- | --- | --- | --- | --- |
| mod1 | reference | reference | (Intercept) | 49.91 | 0.54 | 92.05 |
|  | reference | reference | % SNH | 18.54 | 2.35 | 7.89 |
|  | reference | reference | % early MFC | -3.67 | 1.13 | -3.25 |
|  | reference | reference | Drought | -9.36 | 0.26 | -36.67 |
| mod2 | α | max | (Intercept) | 55.49 | 0.51 | 109.11 |
|  | α | max | % SNH | 17.61 | 2.21 | 7.98 |
|  | α | max | % early MFC | -3.35 | 1.06 | -3.16 |
|  | α | max | Drought | -9.58 | 0.24 | -40.73 |
| mod3 | α | min | (Intercept) | 44.19 | 0.54 | 82.19 |
|  | α | min | % SNH | 18.79 | 2.33 | 8.07 |
|  | α | min | % early MFC | -3.02 | 1.12 | -2.70 |
|  | α | min | Drought | -9.13 | 0.26 | -35.51 |
| mod4 | β | max | (Intercept) | 50.86 | 0.48 | 105.57 |
|  | β | max | % SNH | 14.57 | 2.09 | 6.97 |
|  | β | max | % early MFC | -3.78 | 1.01 | -3.75 |
|  | β | max | Drought | -8.52 | 0.22 | -38.64 |
| mod5 | β | min | (Intercept) | 48.64 | 0.64 | 76.47 |
|  | β | min | % SNH | 23.46 | 2.75 | 8.53 |
|  | β | min | % early MFC | -3.24 | 1.32 | -2.45 |
|  | β | min | Drought | -10.62 | 0.31 | -34.49 |
| mod6 | δ | max | (Intercept) | 78.80 | 0.59 | 133.17 |
|  | δ | max | % SNH | 23.03 | 2.56 | 9.00 |
|  | δ | max | % early MFC | 2.35 | 1.23 | 1.91 |
|  | δ | max | Drought | -10.73 | 0.29 | -37.45 |
| mod7 | δ | min | (Intercept) | 33.30 | 0.47 | 70.16 |
|  | δ | min | % SNH | 9.52 | 2.06 | 4.62 |
|  | δ | min | % early MFC | -10.12 | 0.99 | -10.22 |
|  | δ | min | Drought | -7.71 | 0.22 | -35.02 |
| mod8 | ε | max | (Intercept) | 48.60 | 0.52 | 94.17 |
|  | ε | max | % SNH | 18.42 | 2.23 | 8.24 |
|  | ε | max | % early MFC | -3.14 | 1.08 | -2.92 |
|  | ε | max | Drought | -9.12 | 0.24 | -37.22 |
| mod9 | ε | min | (Intercept) | 50.88 | 0.53 | 95.45 |
|  | ε | min | % SNH | 19.09 | 2.31 | 8.27 |
|  | ε | min | % early MFC | -3.19 | 1.11 | -2.87 |
|  | ε | min | Drought | -9.53 | 0.25 | -37.93 |
| mod10 | pw | max | (Intercept) | 50.67 | 0.48 | 105.51 |
|  | pw | max | % SNH | 15.42 | 2.08 | 7.41 |
|  | pw | max | % early MFC | -3.55 | 1.00 | -3.54 |
|  | pw | max | Drought | -8.70 | 0.22 | -38.70 |
| mod11 | pw | min | (Intercept) | 49.16 | 0.60 | 81.47 |
|  | pw | min | % SNH | 21.25 | 2.61 | 8.14 |
|  | pw | min | % early MFC | -3.86 | 1.26 | -3.07 |
|  | pw | min | Drought | -10.22 | 0.29 | -34.97 |
| mod12 | κ | max | (Intercept) | 49.94 | 0.53 | 94.17 |
|  | κ | max | % SNH | 18.27 | 2.30 | 7.95 |
|  | κ | max | % early MFC | -3.68 | 1.11 | -3.33 |
|  | κ | max | Drought | -9.34 | 0.25 | -37.66 |
| mod13 | κ | min | (Intercept) | 49.87 | 0.53 | 94.13 |
|  | κ | min | % SNH | 18.31 | 2.29 | 7.98 |
|  | κ | min | % early MFC | -3.40 | 1.10 | -3.08 |
|  | κ | min | Drought | -9.32 | 0.25 | -37.16 |
| mod14 | nest_int | max | (Intercept) | 49.92 | 0.53 | 94.92 |
|  | nest_int | max | % SNH | 17.89 | 2.28 | 7.85 |
|  | nest_int | max | % early MFC | -3.32 | 1.10 | -3.03 |
|  | nest_int | max | Drought | -9.28 | 0.25 | -37.49 |
| mod15 | nest_int | min | (Intercept) | 50.17 | 0.52 | 97.16 |
|  | nest_int | min | % SNH | 16.88 | 2.24 | 7.55 |
|  | nest_int | min | % early MFC | -3.66 | 1.08 | -3.40 |
|  | nest_int | min | Drought | -9.26 | 0.24 | -38.02 |
| mod16 | φ | max | (Intercept) | 49.96 | 0.56 | 89.81 |
|  | φ | max | % SNH | 17.78 | 2.41 | 7.37 |
|  | φ | max | % early MFC | -3.40 | 1.16 | -2.93 |
|  | φ | max | Drought | -9.33 | 0.26 | -36.10 |
| mod17 | φ | min | (Intercept) | 50.17 | 0.54 | 93.40 |
|  | φ | min | % SNH | 17.09 | 2.33 | 7.34 |
|  | φ | min | % early MFC | -3.88 | 1.12 | -3.46 |
|  | φ | min | Drought | -9.31 | 0.25 | -37.14 |
| mod18 | μ | max | (Intercept) | 50.01 | 0.51 | 98.67 |
|  | μ | max | % SNH | 17.83 | 2.19 | 8.13 |
|  | μ | max | % early MFC | -3.72 | 1.05 | -3.53 |
|  | μ | max | Drought | -9.35 | 0.25 | -38.09 |
| mod19 | μ | min | (Intercept) | 49.52 | 0.58 | 86.07 |
|  | μ | min | % SNH | 19.23 | 2.50 | 7.70 |
|  | μ | min | % early MFC | -2.78 | 1.20 | -2.31 |
|  | μ | min | Drought | -9.51 | 0.26 | -36.23 |
| mod20 | ψ | max | (Intercept) | 48.41 | 0.42 | 115.34 |
|  | ψ | max | % SNH | 12.45 | 1.82 | 6.85 |
|  | ψ | max | % early MFC | -3.62 | 0.87 | -4.14 |
|  | ψ | max | Drought | -8.28 | 0.20 | -41.40 |
| mod21 | ψ | min | (Intercept) | 51.00 | 0.63 | 81.12 |
|  | ψ | min | % SNH | 23.18 | 2.72 | 8.53 |
|  | ψ | min | % early MFC | -2.34 | 1.31 | -1.79 |
|  | ψ | min | Drought | -10.75 | 0.30 | -35.32 |

Table S8. Likelihood ratio tests for the model predictors SNH, early MFC and drought for the regression analysis.

| Output | Variable | Df | Chisq | P-value |
| --- | --- | --- | --- | --- |
| PS | SNH | 2 | 148.47 | 5.75e-33 |
|  | early MFC | 4 | 33.35 | 1.01e-06 |
|  | Drought | 4 | 256.61 | 2.46e-54 |
| WMax | SNH | 2 | 84.71 | 4.02e-19 |
|  | early MFC | 4 | 99.80 | 1.08e-20 |
|  | Drought | 4 | 375.61 | 5.17e-80 |
| QT | SNH | 2 | 57.45 | 3.35e-13 |
|  | early MFC | 4 | 27.87 | 1.33e-05 |
|  | Drought | 4 | 441.29 | 3.32e-94 |
